# Regulation of TS-1 Zeolite with Small Particle Size by Colloidal Silicon Seed-Induced Synthesis and Application in Oxidative Desulfurization

**DOI:** 10.3390/ma17235722

**Published:** 2024-11-22

**Authors:** Tieqiang Ren, Yue Sun, Yujia Wang, Lulu Wang, Qian Yu, Lisheng Liang, Xianming Kong, Haiyan Wang

**Affiliations:** 1 College of Chemistry and Chemical Engineering, China University of Petroleum (East China), Qingdao 266555, China; tqr_lnpu_121@126.com (T.R.); fsyzww@126.com (Y.W.); gracewangl@163.com (L.W.); 2 School of Petrochemical Engineering, Liaoning Petrochemical University, Fushun 113001, China; sunyue19791980@126.com (Y.S.); qyu@lnpu.edu.cn (Q.Y.); 3 Petroleum Engineering Research Institute of Petrochina Dagang Oil Field Company, Tianjin 300280, China; 13312041240@163.com

**Keywords:** titanium silicon zeolite, regulation, colloidal silicon seed, desulfurization

## Abstract

The dosages of colloidal silicon seeds in the seed-induced synthesis of TS-1 zeolites were investigated in detail. The characterization results revealed that the colloidal silicon seeds not only reduced the particle sizes but also promoted the incorporation of titanium atoms into the framework of TS-1 zeolites as prepared. SEM images and particle size distribution (PSD) confirmed that the particle sizes of TS-1 zeolite could be effectively reduced to about 150 nm. The lattice plane [2 1 0] and [0 2 0] of 7.0-Seed-TS-1 zeolite were well exposed, as observed by the HRTEM images. It is worth noting that the ratio of non-framework Ti atoms incorporated onto the surface of TS-1 zeolites increased slightly to 0.11% by XPS. By regulating the dosage of colloidal Si seeds and promoting rapid nucleation, the size of the crystals could be easily tuned, and then the resulting high external specific surface area and pore volume ensured the reactant accessibility to the active site. The TS-1 zeolites regulated by the 5.0~7.0% dosages of colloidal silicon seeds possessed high external specific surface areas (148.1 m^2^/g and 130.9 m^2^/g) and small particle sizes (about 150 nm). The oxidative desulfurization of 500 ppm DBT by 7.0-Seed-TS-1 zeolite could reach to 100%.

## 1. Introduction

The oxidation of organic molecules to oxygenated chemical compounds plays an important role in bulk and fine chemicals, pharmaceutical, and petrochemical industries. These oxygenated organic molecules are still mainly synthesized through stoichiometric reactions with inorganic oxidants [1]. In the field of petroleum and petrochemicals, the fine desulfurization of fossil fuels has been of concern as the environmental pollution problem [2,3]. It is well known that toxic sulfur dioxide will be produced by the combustion of liquid transportation fuels with a high sulfur content, which is the main source of haze or acid rain.

Compared with hydrodesulfurization technology requiring high temperatures and pressures, oxidative desulfurization (ODS) is a promising technology to obtain oil product with lower sulfur contents due to the high desulfurization efficiency and mild reaction conditions. TS-1 with an MFI structure was first reported from the ENI Company in 1983 [4]. In a previous report, TS-1 showed excellent activity and selectivity in the oxidation of several hydrocarbons such as the epoxidation of alkenes, the hydroxylation of aromatics, the ammoximation of ketones, and the oxidation of bulky organosulfur compounds under mild reaction conditions [5,6,7,8,9,10].

In catalytic oxidation desulfurization, boosting the diffusional efficiency of bulky organosulfur compounds such as dibenzothiophene (DBT) or 4,6-dimethyldibenzothiophene (4,6-DMDBT) and improving their accessibility to the active site of reaction located in the channels or on the surface of zeolite are important for promoting the catalytic performance [11]. In recent years, considerable studies have focused on exploring zeolites with a hierarchical pore structure and Ti enrichment on the surface to reduce the limitations of reactants and products in catalytic reactions [12,13,14,15,16]. The preparation of mesoporous TS-1 zeolites is an attractive research area, with the reported synthesis of mesoporous TS-1 using carbon nanoparticles as hard templates [17]. Lin et al. prepared a TS-1 molecule sieve with large open hierarchical pores through the process of dissolution and re-crystallization, and the introduction of carbon black as a hard template agent, that resulted in a large number of defects in the crystal [18]. The commercial polyvinyl alcohol (PVA) was used as a mesoporous template for preparing TS-1 zeolite with a hierarchical porous structure, in which the three-step crystallization method was included in the process and the product showed excellent catalytic performance in the oxidation reaction of DBT and 4,6-DMDBT [19]. The mesoporous surfactants such as P123 and PS215-PEO100 were introduced and were easily expelled for the preparation of hierarchical pores [20]. In one study, the commercial polymer poly-diallyldimethylammonium chloride (PDADMAC) was used as a dual-functional template to synthesize single-crystalline zeolite with inter-connected mesopores [21,22]. So far, hierarchical TS-1 zeolites have been successfully synthesized by different synthesis strategies, including hard-template and soft-template routes [6,23,24,25,26], steam leaching, and chemical treatment [27,28]. These constructive methods do not mention the effect of zeolite particle size on catalytic performance. The seed-induced method could promote nucleation and accelerate the crystallization rate, resulting in a decrease in crystal size and reducing the amount of the organic structure-directing agent [29]. Zhang et al. studied the tailored synthesis of ultrathin ZSM-5 nanosheets with a controllable b-axis thickness by adjusting the amount and aging time of silicalite-1 seed suspension in the fluoride medium [30]. The thinner nanosheets exhibit better catalytic performance, which can be attributed to their larger surface area and shorter straight channel length.

In this work, TS-1 zeolites with a small particle size were synthesized by the colloidal silicon seed-induced method and the dosages of colloidal silicon seeds were investigated in detail. TS-1 zeolites with different particle sizes could be finely regulated in the range of 150 nm to 250 nm. The characterization results showed that the seeds not only decreased the particle size of TS-1 zeolite but also promoted the incorporation of titanium atoms into the skeleton of TS-1 zeolites. Notably, the TS-1 zeolites that were prepared via the seed-induced method possessed a hierarchical pore structure, a large external specific surface area and an abundant amount of mesoporous volume. Considering the diffusion limitation of reactants and products, TS-1 zeolites as prepared should be used as catalysts with promising applications.

## 2. Materials and Methods

### 2.1. Reagents and Chemicals

Tetraethyl orthosilicate (TEOS, 98%, Shanghai Macklin Biochemical Co., Ltd., Shanghai, China), tetrapropylammonium hydroxide (TPAOH, 25%, Beijing InnoChem Science & Technology Co., Ltd., Beijing, China), tetrabutyl titanate (TBOT, 98%, Guangfu Fine Chemical Research Institute, Tianjin, China), noctane (95%, Shanghai Macklin Biochemical Co., Ltd.), tert-butylhydroperoxide (TBHP, 65 wt% aqueous solution, Sinopharm Group Chemical reagent Co., Ltd., Beijing, China), dibenzothiophene (DBT, 98%, TCI Shanghai Development Co., Ltd., Shanghai, China), and ethanol (100%, Tianjin Wind Boat Chemical Reagent Technology Co., Ltd., Tianjin, China) were used.

### 2.2. Synthesis of x-Seed-TS-1 Zeolite

A measure of 25 g of TEOS was added to 35.15 g of TPAOH (25%) solution and stirred at 35 °C for 6 h to obtain a hydrolyzed transparent solution, and then the mixture was heated to 45 °C for 2 h to remove the ethanol brought by TEOS hydrolysis. Finally, the solution was transferred into a Teflon-lined stainless-steel autoclave and crystallized at 70 °C for 72 h to obtain clarified colloidal silicon seeds.

The starting gel of SiO_2_:TiO_2_:TPAOH:H_2_O:EtOH with a molar ratio of 30:1:12:3600:120 was prepared. Typically, 5.192 g of TPAOH (25%) was mixed with 34.46 g of deionized water at 35 °C, and then the seed of *x* % was added and the mixture was stirred for another 15 min (where *x* was defined as the mass fraction value of seeds in the gel system, %). A measure of 3.32 g of TEOS was added under stirring for another hour. Finally, the mixture of TBOT and ethanol (0.181 g/2.94 g) was added into the solution under severe stirring for 2 h. The starting gel was transferred into a 100 mL Teflon-lined stainless-steel autoclave. After crystallization at 170 °C for 48 h, the solid product was centrifuged twice, rinsed thoroughly with deionized water, and dried at 80 °C for 24 h [31]. Then, the product was calcined at 550 °C for 5 h in an air condition, and the *x*-Seed-TS-1 zeolite was obtained. The value of *x* was 0.1, 0.5, 1.0, 5.0, and 7.0, respectively.

### 2.3. Characterizations

The X-ray diffraction (XRD) spectra were obtained on a Bruker (Bruker Corporation, Karlsruhe, Germany) D8 Advance diffractometer equipped with Cu Kα (λ = 1.5418 Å, 40 kV, 40 mA). Scanning electron microscope (SEM) images were measured on a SU8010 electron microscope (Hitachi Ltd., Tokyo, Japan) with an accelerating voltage of 3 kV. Particle size distributions (PSDs) were detected on Malvern Nano-ZS90 (Malvern Panalytical, Malvern, UK). Transmission electron microscopy (TEM) images and high-resolution (HRTEM) images were collected on a JEM 2100F electron microscope (JEOL Ltd., Tokyo, Japan) with an accelerating voltage of 200 kV. Infrared (FT-IR) spectra were collected on a Nicolet iS50 infrared instrument with the KBr compression method in the range of 400 cm^−1^ to 4000 cm^−1^. The UV-Vis diffuse reflectance spectra (DRS) were recorded in the range between 200 nm and 400 nm on an Agilent Cary5000 spectrophotometer (Agilent Technologies Inc., Sa. Clara, CA, USA). N_2_ adsorption–desorption experiments were performed with an automated gas sorption analyzer of Quantachrome autosorb iQ_2_ (Quantachrome Instruments, Sa. Monica, CA, USA). ESCALAB 250Xi+XPS spectroscopy (Thermo Fisher Scientific, Manchester, UK) was employed to obtain the core-level information of zeolite under the test condition of passing energy of 20 ev, the residence time of 50 ms, the step length of 0.05 ev and 20 runs.

### 2.4. Catalytic Performance Tests

The model fuel with the concentration of 500 ppm of sulfur was prepared by dissolving a certain amount of DBT. The ODS reaction was studied in a conical flask (50 mL) equipped with a ball condenser. In a typical run, 0.0281 g of TBHP served as the oxidant and 0.028 g of octadecane serving as the internal standard substance were dissolved in 10 mL of model fuel, and then 50 mg of *x*-Seed-TS-1 zeolite was added. The reaction was carried out at 65 °C for 135 min. Gas chromatography–mass spectrometry (GC-MS, Agilent 7890A/5975C, equipped with a HP-5MS column, 30 m × 250 µm × 0.25 µm) was utilized to analyze the reaction.

The conversion of DBT was calculated by:

$$\mathrm{Conversion}\;\mathrm{of}\;\mathrm{DBT}\;\left(\%\right)=\frac{n_{\mathrm{DBT}}^{0}-n_{\mathrm{DBT}}}{n_{\mathrm{DBT}}^{0}}\times 100\%$$

The TON (moles of converted DBT per mole of Ti site) of ODS was defined as follows:

$$\mathrm{TON}=\frac{n_{\mathrm{DBT}}^{0}-n_{\mathrm{DBT}}}{n_{\mathrm{Ti}}}\times 100\%$$

## 3. Results and Discussions

### 3.1. Morphology and Characterization of x-Seed-TS-1 Zeolites

**Figure 1:**
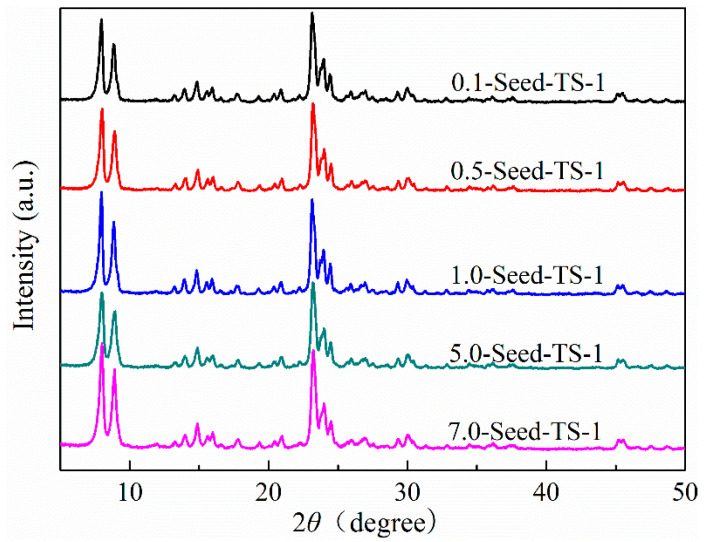
XPD powder patterns of *x*-Seed-TS-1 zeolites.

The XRD patterns of *x*-Seed-TS-1 zeolites were measured and shown in Figure 1. Four intense diffraction peaks were observed at 7.9°, 8.8°, 23.1°, and 23.9°, which were assigned to the typical MFI structure of TS-1 zeolites (PDF#01-070-6302) [32,33]. Their was no diffraction peak observed at 25.4°, which indicated the absence of the TiO_2_ with an anatase phase or its high dispersion due to fine grains. The TS-1 zeolites obtained by colloidal silicon seed-induced synthesis had good phase purity. The diffraction peaks of *x*-Seed-TS-1 zeolites showed high intensity and had approximate crystal sizes of 35.8 nm, 36.1 nm, 38.9 nm, 32.8 nm, and 35.8 nm, as calculated by the Scherrer formula.

**Figure 2:**
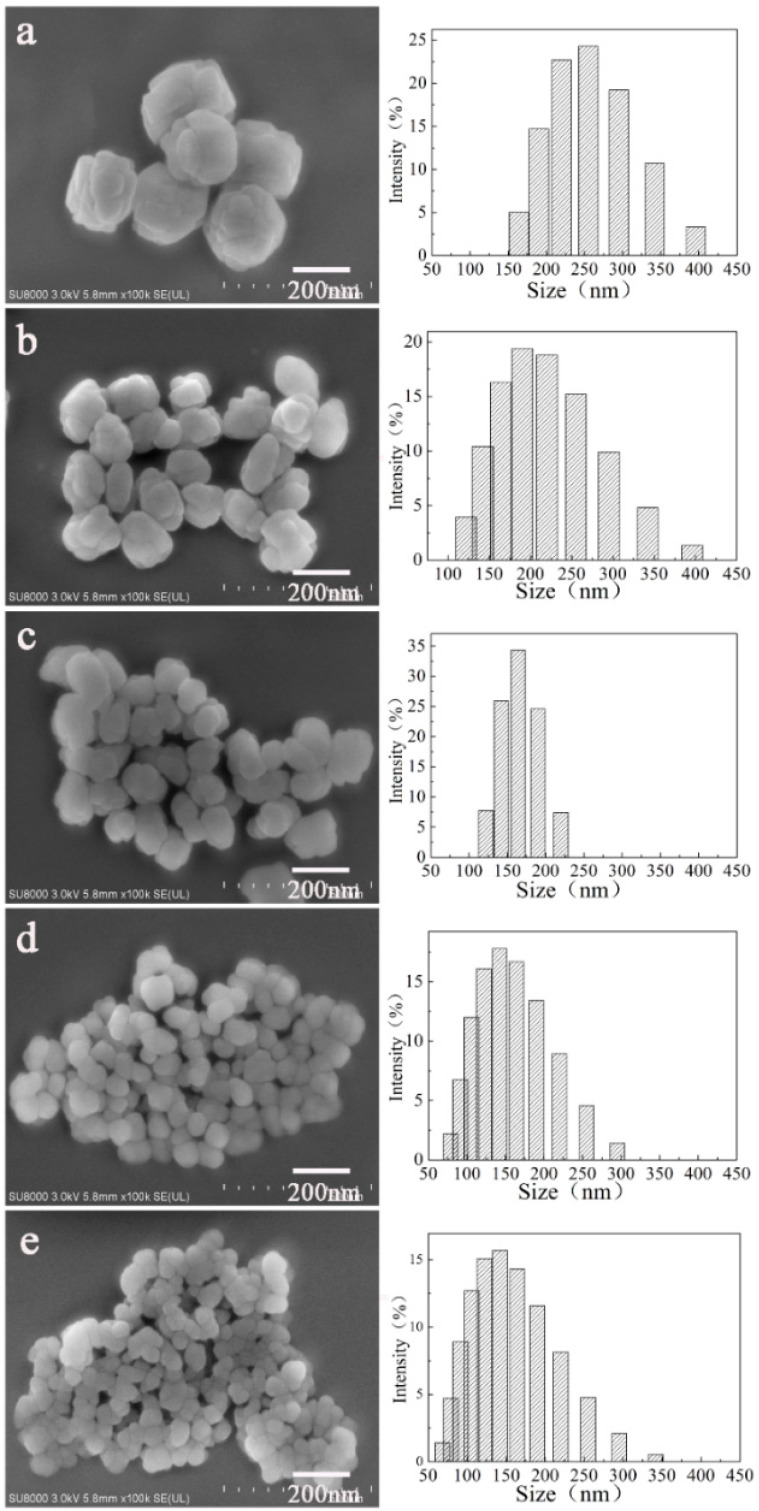
SEM images and particle size distribution of *x*-Seed-TS-1 zeolites: (**a**) 0.1-Seed-TS-1; (**b**) 0.5-Seed-TS-1; (**c**) 1.0-Seed-TS-1; (**d**) 5.0-Seed-TS-1; (**e**) 7.0-Seed-TS-1.

The morphology and PSD of *x*-Seed-TS-1 zeolites were shown in Figure 2. The prepared particles presented a rough surface with different sizes. The dosages of colloidal silicon seed had an obvious influence on the PSD. When the dosage of colloidal silicon seed was controlled at 0.1% or 0.5%, the particle size was regulated at 250 nm or 200 nm. When the seed dosage increased to 1.0%, the particle size reduced by 75 nm compared with 0.1-Seed-TS-1 zeolite. Increasing the seed dosage to 5% and 7%, the particle size was nearly at 150 nm. The PSD of TS-1 zerolite depended on its nucleation and growth process, which could be regulated by adding colloidal silicon seeds to promote rapid nucleation and tuning its dosage to produce small crystals. Due to the significant differences in the diffusion lengths of reactants and products onto the active sites of the external surface, the catalytic performance of TS-1 zeolite was significantly affected. Therefore, the small particle size of TS-1 zeolite prepared by colloidal silicon seed-induced synthesis was a efficient method to satisfy the rising requirement of catalytic oxidation activity.

**Figure 3:**
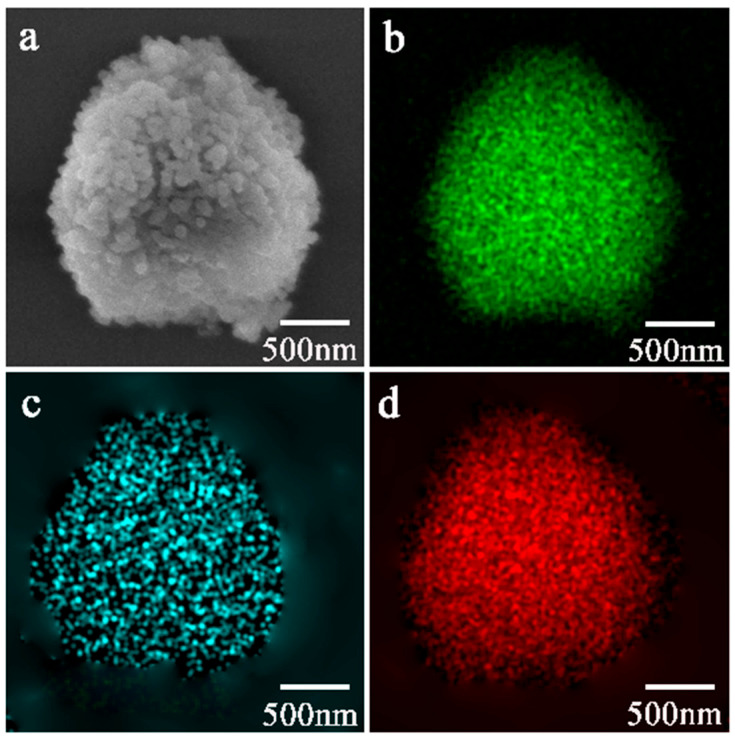
SEM image and element mapping for 7.0-Seed-TS-1 zeolite: (**a**) SEM image for 7.0-Seed-TS-1 zeolite; (**b**) silicon element mapping; (**c**) titanium element mapping; (**d**) oxygen element mapping.

Element mapping for 7.0-Seed-TS-1 zeolite is shown in Figure 3. The 7.0-Seed-TS-1 zeolite had a small particle size of approximately 150 nm and the small particles appeared to aggregate and extrude against each other (Figure 3a). Element mapping results showed that the TS-1 zeolite produced by seed-induced synthesis was composed of Si, Ti, and O elements (Figure 3b–d). The Ti element uniformly dispersed in the interior or on the surface of particles (Figure 3c).

**Figure 4:**
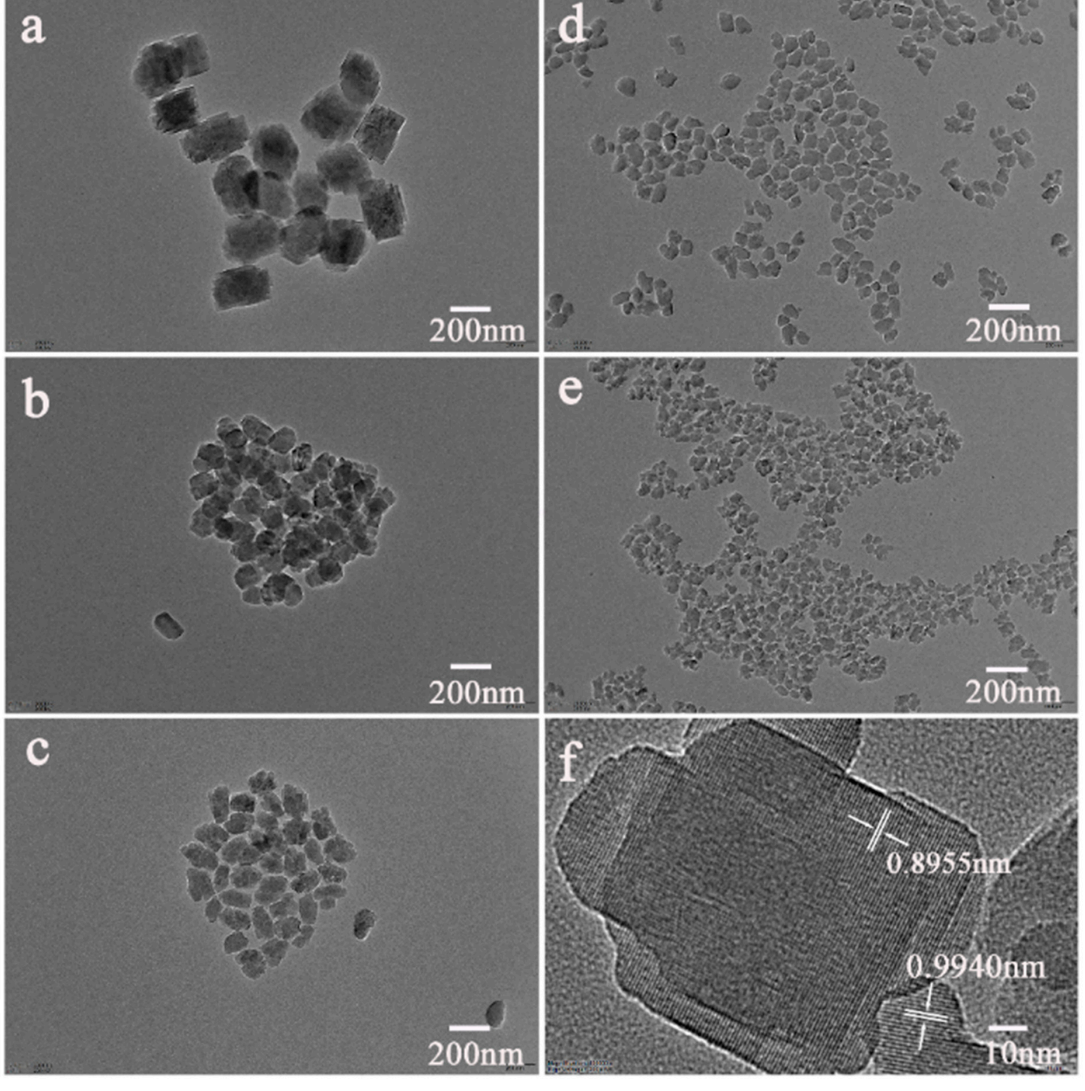
TEM image of *x*-Seed-TS-1 and high-resolution images of (**a**) 0.1-Seed-TS-1; (**b**) 0.5-Seed-TS-1; (**c**) 1.0-Seed-TS-1; (**d**) 5.0-Seed-TS-1; (**e**) 7.0-Seed-TS-1; (**f**) high-resolution image of 7.0-Seed-TS-1.

**Figure 5:**
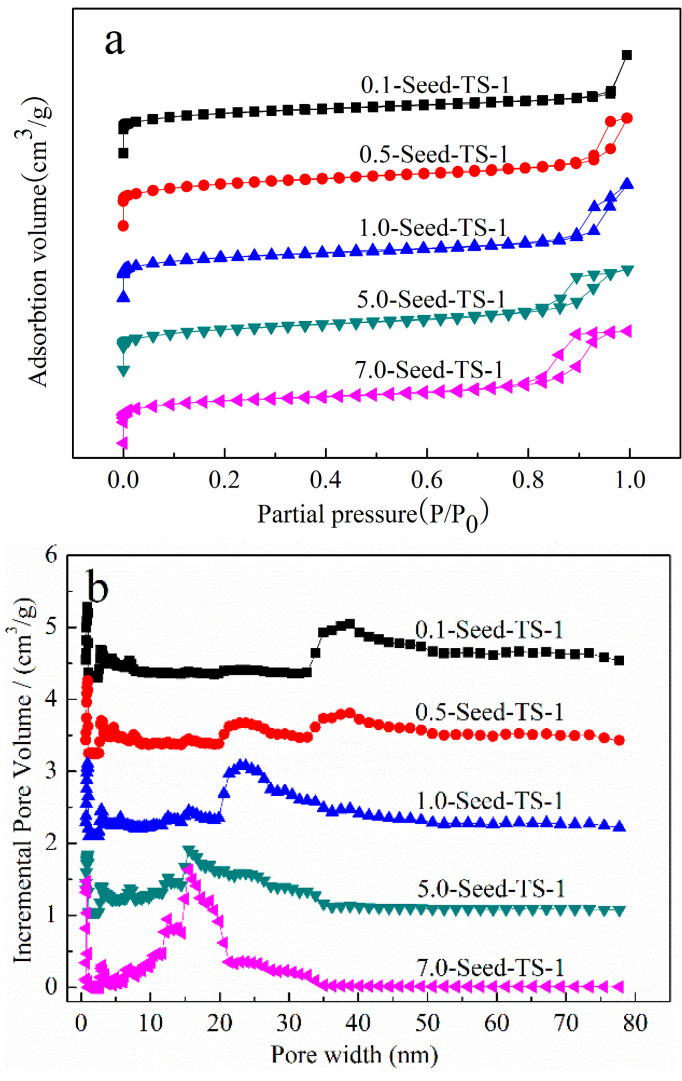
Nitrogen adsorption–desorption analysis of *x*-Seed-TS-1 zeolites: (**a**) adsorption–desorption isotherm; (**b**) pore size distribution by DFT method.

The TEM images of the x-Seed-TS-1 zeolites are shown in Figure 4, where the particle size of x-Seed-TS-1 zeolites prepared by colloidal silicon seed-induced synthesis decreased significantly with the increasing addition of seeds. The 0.1-Seed-TS-1 zeolite had a relatively dispersing particle morphology with a size bigger than 200 nm, which was consistent with the PSD (Figure 2a) and the profile of the particles was obvious. As the addition of seeds to increased to 0.5%, the particle morphology became irregular and the particle size was nearly at 200 nm. As shown in Figure 4a,b, the bright gray areas were due to the hierarchical mesopores inside the 0.1-Seed-TS-1 and 0.5-Seed-TS-1 zeolite particles, which correspond to the 35 nm–45 nm mesopores (Figure 5b). The particle size decreased significantly and particle morphology became more irregular as the addition of colloidal silicon seeds increased. More external surfaces were obtained from 1.0-Seed-TS-1, 5.0-Seed-TS-1, and 7.0-Seed-TS-1 zeolites due to their smaller particle sizes. The high-resolution TEM image of 7.0-Seed-TS-1 zeolite is shown in Figure 4f. There was tow lattice plane spacing at 0.8955 nm and 0.9940 nm corresponding to the lattice plates of [2 1 0] and [2 0 0], respectively. By adjusting the dosage of colloidal silicon seeds, the particle size of TS-1 zeolites could be precisely regulated, and the accessibility of the reactants to the active site was improved due to there being more exposed lattice planes.

The N_2_ adsorption–desorption isotherms of *x*-Seed-TS-1 zeolites were shown in Figure 5a. The five zeolites possessed a typical I adsorption isotherm based on the IUPAC classification, which indicated that the TS-1 zeolites as prepared had a microporous structure feature. When the dosage of colloidal silicon seeds ranged from 0.5% to 7.0%, the curves of adsorption and desorption presented a hysteresis loop between the relative pressure P/P_0_ of 0.8–1.0. This indicated that the condensation of nitrogen gas in the mesopores between the crystals or the mesopores formed by particle aggregation.

The pore distribution based on the cylindrical pore model for *x*-Seed-TS-1 zeolites was calculated using the density functional theory (DFT) method as shown in Figure 5b, in which the intrinsic micropores, hierarchical, and continuous mesopores were obtained. The hierarchical mesopore structure of *x*-Seed-TS-1 zeolites was obviously different by regulating the dosage of colloidal silicon seeds. The mesopore size of 0.1-Seed-TS-1 zeolite was mainly concentrated at 35 nm–45 nm, while the pore size gradually decreased with the increasing dosage of colloidal silicon seeds and was mainly concentrated between 10 nm and 20 nm for 7.0-Seed-TS-1 zeolite. The most obvious mesopore structure between 10 nm and 20 nm was observed from 7.0-Seed-TS-1 zeolite as shown in Figure 5b. The PSD results showed that the particle size decreased significantly from 250 nm to 150 nm. Due to the aggregation of smaller particles, small mesopore structures between 10 nm and 20 nm of 7.0-Seed-TS-1 zeolite could be generated. *x*-Seed-TS-1 zeolites with abundant mesopore channels and enhanced catalytic activity in oxidative desulphurization were mainly ascribed to their small particle size.

It can be seen from texture properties of zeolites that all the *x*-Seed-TS-1 zeolites have a total specific surface of more than 500 m^2^/g (Table 1, column a). Compared with 0.1-Seed-TS-1 zeolite with a microporous structure, the external specific surface area and mesopore volume of other TS-1 zeolites increased significantly (Table 1, columns c and f). Although 0.5-Seed-TS-1 zeolite has a maximum external specific surface area of 160.3 m^2^/g, the particle size was relatively large. It is worth noting that 1.0-Seed-TS-1 and 7.0-Seed-TS-1 zeolites had similar external specific surface areas (134.3 m^2^/g vs. 130.9 m^2^/g) and mesopore volumes (0.4412 cm^−3^/g vs. 0.4269 cm^−3^/g). The increasing addition of seeds could generate an additional specific surface area of approximately 30 m^2^/g for 7.0-Seed-TS-1 zeolite compared to 0.1-Seed-TS-1 zeolite, which was attributed to the promoted nucleation and the accelerating crystallization rate.

7.0-Seed-TS-1 zeolite had the best crystallinity (Figure 1) and generated the highest total specific surface area of 527.9 m^2^/g which was mainly from micropore specific surface area. However, 7.0-Seed-TS-1 has a small particle size at 150 nm (Figure 2e), which could meet the high dispersion requirement of heterogeneous catalytic oxidative desulfurization.

**Figure 6:**
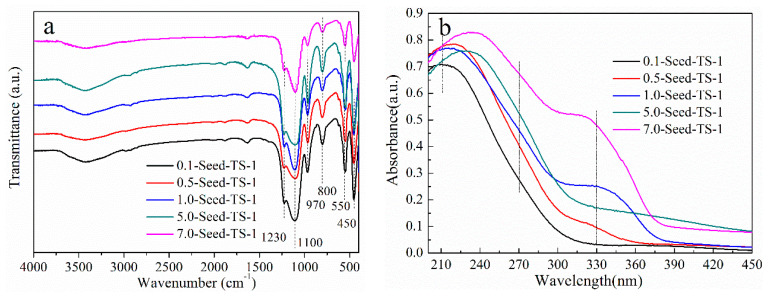
The spectrum analysis of *x*-Seed-TS-1 zeolites: (**a**) FT-IR spectra; (**b**) UV-Vis DRS.

FT-IR spectra found by the KBr pressed-disk technique were used to determine the surface groups of *x*-Seed-TS-1 zeolites (Figure 6a), and six obvious absorption peaks were detected at 450 cm^−1^, 550 cm^−1^, 800 cm^−1^, 970 cm^−1^, 1100 cm^−1^, and 1230 cm^−1^. The absorption peaks at 550 cm^−1^, 800 cm^−1^, and 1230 cm^−1^ were assigned to the stretching vibration mode of the -O-Si-O- and -O-Ti-O- groups belonging to the double five-membered rings [34], which were in accordance with the typical MFI structure of TS-1 zeolites (Figure 1). Ti species were substantially incorporated into the zeolite skeleton, and the obvious peak at 970 cm^−1^ was attributed to the stretching vibration mode of the Si-O-Ti bond or the Si-O bond perturbed by the framework titanium species [35,36]. The relative intensity ratio between 970 cm^−1^ and 800 cm^−1^ in the FT-IR spectrum increased linearly with the increasing of the Ti content in the framework [37]. Therefore, the absorption intensity ratio of I970/I800 was used to determine the relative content of Ti species in the zeolite framework structure, which was 1.15, 1.13, 1.13, 1.10, and 1.07 corresponding to 0.1-Seed-TS-1, 0.5-Seed-TS-1, 1.0-Seed-TS-1, 5.0-Seed-TS-1, and 7.0-Seed-TS-1, respectively. The intensity ratio of I970/I800 for *x*-Seed-TS-1 zeolites was higher than the 0.83 of conventional TS-1 zeolite [38]. It can be concluded that colloidal silicon seed-induced synthesis could promote the incorporation of titanium atoms into the framework of TS-1 zeolite.

The optical density ratio of the 550 cm^−1^ to 450 cm^−1^ band (I550/I450) can be used to understand the crystallization yield of TS-1 zeolites [39]. The optical density ratio of I550/I450 for 0.1-Seed-TS-1, 0.5-Seed-TS-1, 1.0-Seed-TS-1, 5.0-Seed-TS-1, and 7.0-Seed-TS-1 is 0.89, 0.91, 0.89, 0.91, and 0.93, respectively. The value of I550/I450 for *x*-Seed-TS-1 is much higher than that of 0.67 for conventional TS-1 zeolite, and *x*-Seed-TS-1 zeolite shows a good crystallization yield.

The chemical environment of titanium species in *x*-Seed-TS-1 was investigated by UV-Vis DRS (Figure 6b). The *x*-Seed-TS-1 zeolites assisted by colloidal silicon seeds had a strong ultraviolet absorption at 210 nm, which was assigned to the charge transfer between titanium atoms and oxygen atoms in the tetrahedral coordination [40]. It was further confirmed that Ti atoms entered into the zeolite skeleton. It was noted that the absorption peaks of 5.0-Seed-TS-1 and 7.0-Seed-TS-1 zeolites were located in a broadened range, which corresponded to partially polymerized titanium species with a hexahedral coordination at 270 nm [41]. The absorption peak at 330 nm indicated the existence of TiO_2_ in 1.0-Seed-TS-1 and 7.0-Seed-TS-1 zeolites, which was not detected in the XRD (Figure 1). The fine grain of the TiO_2_ phase was highly dispersed in *x*-Seed-TS-1 zeolite’s main phase [42]. It is worth noting that the low dosage of colloidal silicon seeds could inhibit the formation of the TiO_2_ phase. However, the increasing dosage of colloidal silicon seeds for 1.0-Seed-TS-1 or 7.0-Seed-TS-1 generated the TiO_2_ phase. A large number of colloidal silicon seeds was beneficial to adsorb Ti species randomly rather than Si species. The TiO_2_ phase may lead to reduced oxidant utilization, while it imposed no obvious adverse effects on the catalytic activity of *x*-Seed-TS-1 [39].

**Figure 7:**
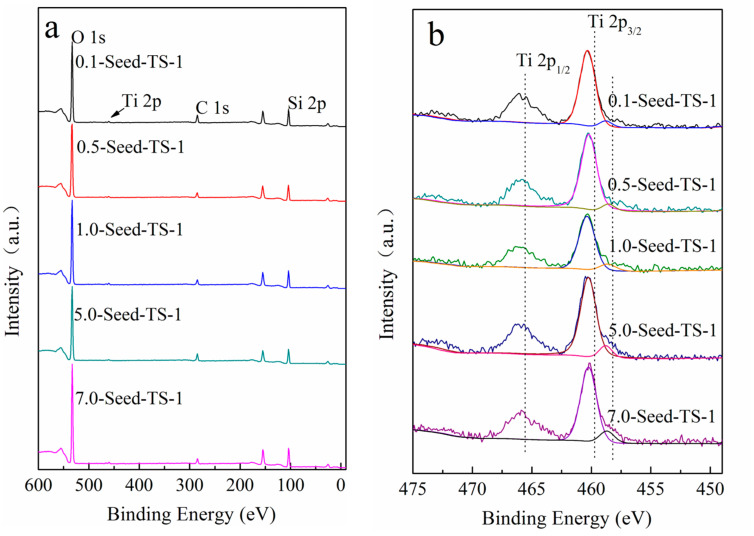
The XPS spectrum of *x*-Seed-TS-1 zeolites: (**a**) full survey XPS spectrum of *x*-Seed-TS-1 zeolites; (**b**)Ti 2p region of *x*-Seed-TS-1 zeolites.

The content and the chemical state of the Ti species on the external surface of *x*-Seed-TS-1 zeolites were investigated by the peak fit analysis of XPS spectroscopy (Figure 7). The full survey XPS spectrum is shown in Figure 7a. It can be detected that there were there main orbitals corresponding to O1s, Ti 2p, and Si2p electrons (Figure 7a). The Ti 2p_3/2_ at 460.2 eV and the Ti 2p_1/2_ at 466.0 eV were assigned to the framework Ti atoms in tetrahedral coordination. The Ti 2p_3/2_ orbital electrons at the binding energy of 460.2 eV split into a new orbital, of which the binding energy was 459.0 eV, indicating the formation of new Ti species in hexahedral coordination (TiO_6_) [37].

Accompanied with the increasing dosage of colloidal silicon seeds, the hexahedral coordination structure on the surface of zeolite also increased slightly. The relative content of non-skeleton Ti atoms on the surface of 7.0-Seed-TS-1 zeolite could reach up to 0.11% (Table 2, column d). Additionally, it had been proven that the activity of the hexahedral coordination exhibited was 2~3 times higher than that of the tetrahedral coordination. The particle size of TS-1 zeolites could be significantly reduced through the colloidal silicon seed-induced synthesis route, which could also attach a Ti hexahedral coordination structure onto the surface.

**Figure 8:**
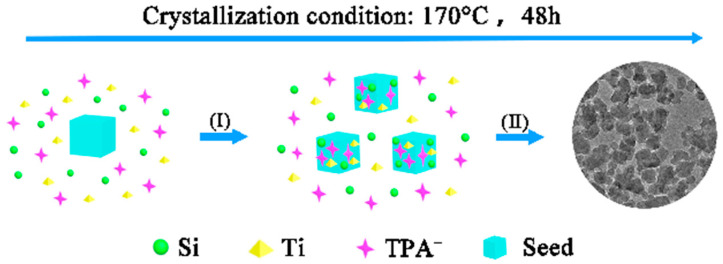
Mechanism of formation of *x*-Seed-TS-1 zeolite.

Here, the mechanism of formation of x-Seed-TS-1 zeolite is mentioned in Figure 8. Firstly, Ti species, Si species, TPA^+^ cations, and colloidal silicon seeds were fully dispersed in the starting gel and interacted with each other. Due to the abundant hydroxyl groups on the surface of colloidal silicon seeds, Ti species, Si species, and TPA^+^ cations were directly adsorbed on the surfaces of seeds. Then, XRD results showed that zeolites prepared by the increasing colloidal silicon seeds exhibited a well-defined reflection at 7.9° (Figure 1). By regulating the dosage of colloidal silicon seeds and promoting rapid nucleation, the size of the crystals can be easily tuned (Figure 8).

### 3.2. Catalytic Performance

The oxidation performance for organic sulfide DBT of the prepared *x*-Seed-TS-1 zeolites was investigated by using TBHP as the oxidant. The reaction conditions are listed in Table 3; the prepared zeolites exhibited excellent catalytic activity in 75 min, and the conversion rate of DBT was continuously increasing. 5.0-Seed-TS-1 and 7.0-Seed-TS-1 zeolites presented high catalytic performance, and the conversion rate of DBT could achieve up to 87.8% and 93.5%, which is much higher than the approximate 85% of TS-1 zeolite [25].

The 7.0-Seed-TS-1 zeolite had good dispersion in a heterogeneous system and ensured the accessibility of organic sulfur compounds and oxidants to the active site of Ti. The TON values were 24.3 and 13.7 from zeolites with 150 nm and 250 nm diameters. It was concluded that the smaller PSD of the catalyst could provide better catalytic activity. The non-skeleton Ti species on the surface of *x*-Seed-TS-1 increased slightly, and they also promoted the catalytic activity in oxidation desulfurization. In 135 min, the conversion rate of DBT on 5.0-Seed-TS-1 and 7.0-Seed-TS-1 zeolites could reach up to 98.6% and 100%, respectively.

**Figure 9:**
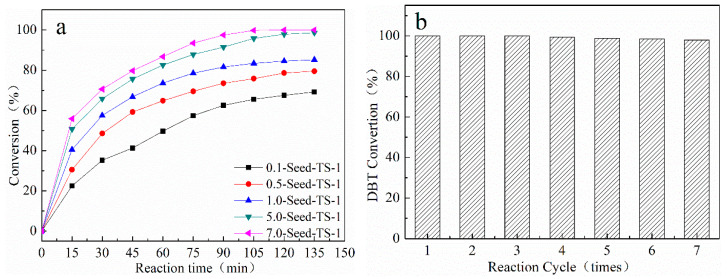
Catalytic performance of *x*-Seed-TS-1 zeolites: (**a**) catalytic oxidation of DBT with TBHP over *x*-Seed-TS-1 zeolites; (**b**) recycling tests in the oxidation of DBT over 7.0-Seed-TS-1 zeolite. Reaction conditions: 10 mL of 500 ppm DBT model fuel, 65 °C, 50 mg of catalysts, n(DBT)/n(TBHP) was 0.5.

The recycling performance of 7.0-Seed-TS-1 zeolite was investigated. The catalyst after the reaction was separated by a high-speed centrifuge and washed three times with ethanol. Then, the catalyst was roasted at 550 °C for 48 h under air conditioning. After seven recycles, the 7.0-Seed-TS-1 zeolite still showed excellent catalytic activity, and the conversion rate of DBT was still 98.3% (Figure 9). Through the catalytic performance investigation of colloidal silicon seed-induced TS-1 zeolites, the seeds could control TS-1 zeolite particle size accurately, and could meet the needs of the high-performance oxidation desulfurization catalyst.

## 4. Conclusions

In summary, we studied the interaction between the dosage of colloidal silicon seeds and the PSD of TS-1 zeolites during the preparation process. By utilizing colloidal silicon seeds in the synthesis of TS-1, the particle size of TS-1 zeolite could easily reach to 150 nm, which resulted in 7.0-Seed-TS-1 zeolite, by unit mass, having a higher dispersing performance than others. The seed-induced synthesis could also promote nucleation and accelerate the crystallization rate as XRD results. At the same time, the *x*-Seed-TS-1 zeolite possessed an abundant external specific surface area and mesoporous pore volume, which could overcome the diffusion limitation of large organic sulfur molecules to active Ti sites. The seed-induced synthesis method also promoted the incorporation of titanium atoms into the skeleton of TS-1 zeolites compared to conventional TS-1. It is concluded that the small particle size, abundant external specific surface, and the Ti atoms incorporated into the skeleton have the synergistic effect on the effective removal of organic sulfur from fuel. Here, the reactive activity of x-Seed-TS-1 zeolites was investigated under the above reaction conditions. In future research, the effect of the temperature in the crystallization step on the final crystal size or the reactive kinetics of organic sulfur removal will be investigated in detail. Seed-induced synthesis is a promising method for a high-performance catalyst.

## Figures and Tables

**Table 1:** The texture properties of *x*-Seed-TS-1 zeolites as prepared.

Sample	$S_{total}^{\;\;a}\;$ (m^2^/g)	$S_{micro}^{\;\;b}$ (m^2^/g)	$S_{external}^{\;\;c}$ (m^2^/g)	$V_{total}^{\;\;d}$ (cm^−3^/g)	$V_{micro}^{\;\;e}$ (cm^−3^/g)	$V_{meso}^{\;\;f}$ (cm^−3^/g)	$d_{\mathrm{average}}^{\;\;g}$ (nm)
0.1-Seed-TS-1	500	381.0	119.0	0.5262	0.1701	0.3561	4.21
0.5-Seed-TS-1	516.7	356.4	160.3	0.5751	0.1604	0.4147	4.452
1.0-Seed-TS-1	508.5	374.2	134.3	0.6063	0.1651	0.4412	4.769
5.0-Seed-TS-1	512.1	364	148.1	0.5411	0.1637	0.3774	4.226
7.0-Seed-TS-1	527.9	397	130.9	0.5987	0.1718	0.4269	4.536

^a^ Specific surface area calculated using the BET equation. ^b^ Micropore area calculated using the t-plot method. ^c^ External surface area. ^d^ Total pore volume at 0.995 of P/P_0_. ^e^ Micropore volume calculated using the t-plot method. ^f^ Mesopore volume resulting from the total pore volume subtracting the micropore volume. ^g^ Average pore diameter calculated by the BET specific surface area and total pore volume of the cylindrical pore model.

**Table 2:** Surface atomic composition by XPS and Ti relative content by FT-IR of *x*-Seed-TS-1 zeolites.

Sample	${Si\;}_{\mathrm{Atomic}\%\;}^{a}$	${Ti\;}_{\mathrm{Atomic}\%\;}^{b}$	$Si/Ti_{\mathrm{Atomic}\;\mathrm{ratio}}^{\;c}$	${\mathrm{Non-Framework}\;\;Ti\;}_{\mathrm{Atomic}\%}^{\;d}$	$I_{970/800}^{\;\;e}$
0.1-Seed-TS-1	33.80	0.46	73.47	0.08	1.15
0.5-Seed-TS-1	33.22	0.50	66.44	0.08	1.13
1.0-Seed-TS-1	33.58	0.52	64.57	0.07	1.13
5.0-Seed-TS-1	32.72	0.42	77.90	0.10	1.10
7.0-Seed-TS-1	33.12	0.45	73.60	0.11	1.07

^a,b,c,d^ Measured by XPS. ^e^ FT-IR intensity ratio of the bands at 970 cm^−1^ and 800 cm^−1^.

**Table 3:** Oxidative desulfurization of DBT with TBHP as the oxidizing agent over *x*-Seed-TS-1 zeolites ^a^.

Sample	Si/Ti ^b^	PSD ^c^ (nm)	Conv. DBT ^d^ (%)	Sel. DBSO_2_^e^ (%)	TON ^f^
0.1-Seed-TS-1	39.96	225–300	57.4	60.8	13.7
0.5-Seed-TS-1	36.69	175–225	69.5	53.6	15.6
1.0-Seed-TS-1	45.05	150–200	78.5	51.3	18.1
5.0-Seed-TS-1	57.70	125–175	87.8	45.3	23.2
7.0-Seed-TS-1	52.04	125–175	93.5	48.5	24.3

^a^ Reaction conditions: 10 mL of 500 ppm model fuel, 65 °C, 50 mg of catalysts, n(DBT)/n(TBHP) was 0.5. ^b^ Measured by Energy Dispersion Spectrum (EDS). ^c^ Measured by particle size distributions (PSDs). ^d^ Conversion of DBT (Conv. DBT) after 75 min of the catalytic reaction. ^e^ Selectivity of DBSO_2_ (Sel. DBSO_2_) based on the DBT after 75 min of the reaction. ^f^ TON: Turnover number (moles of converted DBT per mole of Ti site).

## Data Availability

The original contributions presented in the study are included in the article, further inquiries can be directed to the corresponding author.
